# CpG Islands: Starting Blocks for Replication and Transcription

**DOI:** 10.1371/journal.pgen.1000454

**Published:** 2009-04-10

**Authors:** Marie-Noëlle Prioleau

**Affiliations:** Institut Jacques Monod, CNRS, Université Paris 7, Université Pierre et Marie Curie, Paris, France; Medical Research Council Human Genetics Unit, United Kingdom

The ongoing release of numerous genome sequences constitutes a scientific landmark that has transformed our way of thinking about the organization of living matter. The complete list of annotated genes that is finally at hand pushes us to a stage where we can now explore how genomes harmonize the regulation of gene expression with other processes that must concurrently take place along the genome. A major unresolved question pertaining to mammalian genomes is how their replication is precisely coordinated so that exactly one copy is made at each cell cycle. Despite extensive efforts aimed at understanding the molecular mechanisms involved in the prevention of abnormal replication (i.e., over- or under-replication), the molecular determinants for selecting the starting points (termed “origins,” or ORIs) of replication remain poorly understood [1].

Until recently, only a very small number of ORIs had been mapped, hindering the development of an accurate and global view of origin specification. Indeed, what is true for a specific ORI can be misleading for the majority of ORIs because of the flexibility of mechanisms that are involved in origin selection. As a result, the lack of a statistically relevant dataset has led to precocious and incorrect conclusions. In the past few years, genome-wide studies have been used, at a massive scale, to study transcription and chromatin structure, providing an integrated view of genome organization and gene regulation. Several genome-wide studies focusing on the analysis of DNA replication timing have shown a global coordination between gene regulation and replication timing, with GC-rich regions replicating early and AT-rich replicating late [2],[3]. Moreover, a recent study showed that 20% of the mouse genome displayed important changes in replication timing upon differentiation of embryonic stem (ES) cells, suggesting a highly dynamic regulation [4]. Until very recently, however, large-scale mapping of replication origins had been prevented by the technical difficulties inherent to obtaining enough samples with good enrichment in ORI sequences. A few months ago, common features shared by large groups of ORIs were extracted from a large-scale mapping of replication origins based on the resistance of short nascent strands (SNS) to λ-exonuclease digestion [5]. This study, conducted in HeLa cells and covering approximately 1% of the human genome, showed that origin density is strongly correlated with genomic landscapes, with clusters of closely spaced origins in GC-rich regions and no or only few origins in GC-poor regions. Moreover, half of the origins were mapped within or near CpG islands.

In this issue of *PLoS Genetics*, a study by Sequeira-Mendes et al. extends this observation to ORIs in mouse ES cells and shows the conservation of origin specification mechanisms across species and cell types [6]. In this study, Sequeira-Mendes et al. also explored the relative strength of mapped ORIs. Indeed, in contrast to the first study, in which amplified SNS were hybridized on microarrays, Sequeira-Mendes et al. used nonamplified SNS extracted from a large population of cells, giving them semi-quantitative information on ORI efficiency. They observed that, in general, origins that lie within CpG island promoter are more efficient than non-CpG origins. They confirmed this result on a subset of origins by quantitative analysis in ES cells and fibroblasts. Another strength of this study is the use of short-size SNS, allowing the authors to precisely localize ORIs. They observed a strong correlation between the 5′ end of annotated genes and highly efficient ORIs. This correlation was especially remarkable in the case of promoters containing alternative transcription initiation sites: here, distinct points of replication initiation were located immediately adjacent to the mapped transcription start sites. This result raises the question of whether the spatial coincidence between replication and transcription initiation has a functional significance. It remains to be seen whether the transcriptional and replication machineries recognize similar features. However, it is also important to point out that in both studies, a significant fraction of ORIs are associated neither with known promoters nor with histone modifications indicative of transcriptional activity. Therefore ORI specification, at least for a subset of origins, is not tightly linked to transcription. This subtype of origins could possibly share molecular mechanisms with highly efficient CpG island promoter origins for origin specification. Alternatively, they might have developed another strategy to trigger replication initiation with lower efficiency.

Origin sequences found in human cells are evolutionarily conserved among mammals, suggesting the maintenance of specific *cis*-elements at replication origins [5],[7]. The identification of a large collection of origins now gives the opportunity to search for specific over-represented sequence motifs using computational tools for de novo prediction of regulatory elements. Such studies should provide new information on origin selection mechanisms. It should be noted that methods used in both studies were designed to identify focused origins. Although these studies could detect weak origins of replication active in only 10% of cell cycles, the sensitivity of the analyses was not sufficient to explore previously described replication initiation zones [8],[9]. The development of new methods based on deep sequencing associated with powerful statistical analysis should allow not only origin mapping along entire genomes, but also the search for broad zones of initiation. Such regions should have, along the zone of initiation, a signal of SNS enrichment notably higher than in domains devoid of origins.

Finally, Sequeira-Mendes et al. found that ORIs located within promoters are significantly enriched in genes that are expressed during early development. They proposed that this marks the competence to drive replication during the rest of development. Whole-genome analyses in different cell lines and across several species will allow testing of this hypothesis with a larger dataset. This will also provide information on the dynamics of evolution of replication origins in vertebrates and enable researchers to investigate the constraints imposed by replication on genome organization, a topic that is a matter of debate in the community [7]. Finally, analyses of origin firing at specific loci by in vivo labelling and single-molecule analysis by DNA combing should help to complement and confirm whole-genome approaches [10]–[12]. The future development of these methods will ultimately give a vision that hews closely to the true complexity of origin firing in mammalian genomes.
